# Would You Consider Accepting a Pig Kidney Transplant?

**DOI:** 10.34067/KID.0000000646

**Published:** 2025-01-30

**Authors:** Patrick O. Gee

**Affiliations:** 1 Healthcare Consultant/Patient Advocate, iAdvocate, North Chesterfield, Virginia

**Keywords:** CKD, diabetes, dialysis access, transplantation

When I ask, “Would you accept a pig kidney transplant?” I reflect on my journey with kidney disease. Diagnosed with type 2 diabetes in 2003, I learned in 2013 that I had stage 3b ESKD, leading me to start peritoneal dialysis later that year.

I underwent a kidney transplant on April 21, 2017, which became a nightmare with delayed graft function, blood clots, and a 33-day hospital stay. After many challenges, my new kidney finally functioned on day 47. This makes me wonder if a pig kidney would have changed my experience.

As a health care consultant and patient advocate, I see the urgent need for alternatives such as xenotransplantation. The demand for human kidneys far exceeds supply, making genetically modified pig kidneys a potential solution. Here are four key points to consider.

## Safety Concerns

### Genetic Modification

Pigs are often changed genetically to reduce the risk of organ rejection and improve compatibility with human recipients, thereby addressing immune response concerns.

### Immune Response Management

Despite promising modifications, questions about the human immune system's reaction to foreign organs need ongoing research.

### Infectious Disease Risks

Although pigs may carry harmless viruses, their potential risks to humans cannot be ignored. Rigorous screening and monitoring processes are essential to mitigate these dangers.

### Long-Term Outcomes

We must prioritize further investigation into the durability and functionality of pig kidneys over time.

### Ethical and Psychological Factors

The ethical implications of xenotransplantation demand immediate attention because they pertain to our moral obligations toward human and animal life. This emerging medical field raises critical questions about the sanctity of life and the alteration of the natural order. In addition, we must consider the psychological effects on recipients who may face challenges in accepting an organ from an animal. Addressing these concerns is essential for fostering an environment where ethical practices are aligned with psychological support, thus ensuring comprehensive patient care. This approach advances medical science and reinforces our commitment to compassion and integrity in health care.

### Regulatory Oversight

A thorough regulatory framework is essential to ensure the safety and efficacy of xenotransplantation practices.

## Risks and Concerns

### Infection Risks

Acknowledging the potential danger posed by pig-specific pathogens that may be transmitted to humans is crucial. Although pigs are raised in controlled environments designed to mitigate these risks, we cannot ignore the substantial threat they still pose. We must remain vigilant and proactive in addressing this issue to protect public health.

### Immune Rejection

Despite advancements, the risk of immune rejection stays a pressing concern. The human immune system may recognize the pig kidney as foreign and mount an immune response against it, leading to organ rejection. This can involve antibody-mediated rejection, where the body produces antibodies that attack the transplanted organ.

### Ethical Dilemmas

The moral implications of using animal organs for human transplants demand thoughtful consideration.

### Long-Term Viability

We need to investigate the long-term outcomes of pig kidneys in human recipients. Early evidence shows that they can perform essential functions, but uncertainties about their durability remain. It is crucial to thoroughly study how well these organs can mimic human kidney functions over time. By prioritizing this research, we can advance organ transplantation solutions and improve the quality of life for many needy individuals. Let us champion this exploration for health and hope!

## Addressing Emotional and Ethical Complexities

Although the promise of an endless supply of organs is enticing, the decision to accept a pig kidney is not without its challenges. As an ordained elder and a patient advocate, I recognize the weight of cultural and spiritual considerations. Many traditions view the use of pig products with skepticism or prohibition, which complicates the decision-making process. Balancing the potential for life-saving treatment with deeply held beliefs presents a profound ethical dilemma.

## Potential Benefits

### Addressing Organ Shortages

Pig kidney transplants could significantly alleviate the dire shortage of human organs, potentially saving countless lives.

### Advancements in Genetic Engineering

Innovations in genetic modification aim to reduce rejection risks and enhance compatibility with human recipients.

### Functional Promise

Early studies show that genetically modified pig kidneys can function effectively within human bodies, providing hope to patients with limited treatment options.

## Recent Breakthroughs in Organ Transplantation

### A Historic Milestone

#### The First Successful Transplant

On March 21, 2024, a remarkable achievement marked a turning point in medical history. The world's first successful transplant of a genetically edited pig kidney was performed on a 62-year-old man suffering from end stage kidney disease by an innovative team at Harvard. This landmark procedure highlights human ingenuity and ignites hope for countless individuals waiting for organ transplants. The future of organ transplantation is bright, and we must embrace this opportunity.

### Proven Functionality and Promising Trials

Before this monumental event, researchers at New York University's Langone Health proved that a genetically engineered pig kidney functioned exceptionally well for 32 days in a brain-dead human recipient. This success paves the way for further exploration into the viability of pig kidneys as a life-saving solution for those in dire need.

### A Future of Possibilities

Ongoing research yields positive outcomes, proving that genetically modified pig kidneys can keep human subjects functional. Each successful trial brings us closer to understanding such transplants' long-term implications and safety. The prospect of a reliable organ source could revolutionize transplantation, ensuring that patients receive the care they desperately need.

## My Closing Reflection

Considering the implications of accepting a pig kidney involves deeply personal medical decisions, influenced by individual beliefs and values. This choice goes beyond medicine; it navigates ethical and spiritual landscapes that require empathy.

As we honor patients' desires for health and dignity, I will personally consider a pig kidney if my kidneys fail, and advancements continue.

It is time to engage in thoughtful dialog about xenotransplantation, embracing innovation while critically assessing its effects. Together, we can work toward a future where no patient is left waiting for life-saving options and advocate for health equity and patient empowerment.

## Supplementary Material

