# Supplementary material for: Would You Consider Accepting a Pig Kidney Transplant?
Source: Kidney360. 2025 Jan 30;6(1):150–1. doi: 10.34067/KID.0000000646 (PMC11793191; doi:10.34067/KID.0000000646)
Supplement: Supplementary file 1 [file kidney360-6-150-s001.pdf]

ASN American Society of Nephrology | JASN Journal of the American Society of Nephrology | KIDNEY360 Accessing Our World From Every Angle | CJASN Clinical Journal of the American Society of Nephrology

ASN Journal Disclosure Form

As per ASN journal policy, I have disclosed any financial relationships or commitments I have held in the past 36 months as included below. I have listed my Current Employer below to indicate there is a relationship requiring disclosure. If no relationship exists, my Current Employer is not listed.

P. Gee reports the following:
Employer: Self-Employed; iAdvocate, Inc., Health & Wellness organization; Self-employed; P Gee Consulting, LLC.; Consultancy: FOUNTAIN EAC (Bayer International); Boehringer Ingelheim ; Virginia Commonwealth University (VCU); Evidation Health, Walgreen, The PATIENTS Professors Academy, Univ. Of Maryland-Baltimore,; Honoraria: Patient Family Advisors Network (PFA Network), APOLLO APOL1 Long-term Kidney Transplantation Community Advisory Council (CAC), CareDX, Bayer International, Vertex International, NephCure International, AMGEN, Traverse, and Patient Family Center Care Partners (PFPC partners); American Kidney Fund, FOUNTAIN (Bayer International). Boehringer Ingelheim, Robert Woods Johnson Foundation, VCU, American Kidney Fund, Boehringer-Ingelheim, and PxP.; Advisory or Leadership Role: KHI Patient Family Partnership Council Chair; KHI Strategy BOD Member; and Organ Procurement Transplant Network Kidney Transplantation Committee member, National Kidney Foundation Health Equity Advisory Board; FOUNTAIN Executive Advisory Committee (Bayer International), AHA Cardio-Kidney Metabolic Health Patient Advisory Group, Walgreen?s Patient Advisory Board, PATIENTS Professor Academy?s Steering Committee, Walgreen?s Patient Advisory Group; Speakers Bureau: Boehringer Ingelheim; and Other Interests or Relationships: AKF Ambassador and Kidney Health Coach; NKF KAC; UNOS Ambassador; PCORI Ambassador ; NCC PFE-LAN SME; KPAC Member; Quality Insights Renal Network 5 PAC Chair; ASN Diabetic Kidney Disease-Collaborative Task Force, CareDX Ambassador, ESRD Health Equity Advisory Board, National Kidney Foundation Spring Clinical Meeting Planning Committee, and KHI APOL-1 Steering Committee, and AKHOMM Workgroup.

I understand that the information above will be published within the journal article, if accepted, and that failure to comply and/or to accurately and completely report the potential financial conflicts of interest could lead to the following: 1) Prior to publication, article rejection, or 2) Post-publication, sanctions ranging from, but not limited to, issuing a correction, reporting the inaccurate information to the authors' institution, banning authors from submitting work to ASN journals for varying lengths of time, and/or retraction of the published work.

Name: Patrick O. Gee
Manuscript ID: K360-2024-000958
Manuscript Title: Patient Perspective: Would You Consider Accepting a Pig Kidney Transplant?
Date of Completion: November 7, 2024
Disclosure Updated Date: November 7, 2024
